# Clinical Conundrum: SSRI Emergent Hypomania, a Turn in the Road to Bipolarity?

**DOI:** 10.1111/bdi.70031

**Published:** 2025-05-03

**Authors:** Matthew Cohen, Prateek Varshney, Jasmine Ronsisvalle, Violeta Perez‐Rodriguez

**Affiliations:** ^1^ Department of Psychological Medicine Institute of Psychiatry, Psychology & Neuroscience, King's College London London UK; ^2^ Child and Adolescent Mental Health Services South London and Maudsley NHS Foundation Trust London UK; ^3^ School of Psychology, Children and Young People's Psychological Training Institute of Psychology, Psychiatry & Neuroscience London UK; ^4^ Department of Psychosis Studies Institute of Psychiatry, Psychology & Neuroscience, King's College London London UK

## 1 Case Presentation

(All identifiable patient information has been anonymised to maintain confidentiality).

Jane is a 16‐year‐old female who presented to child and adolescent mental health services (CAMHS) in June 2023 following 9 months of insidious onset, progressively deteriorating mood and social anxiety. Her symptoms had recently worsened with passive suicidal ideations and increased aggression, in the context of familial disputes and rising academic pressures. Jane had a history of domestic abuse in her early years leading to parental separation. Jane had been previously known to CAMHS and received family therapy at the age of 10 for low mood in the context of bullying. Jane's parent and younger sibling had DiGeorge syndrome. Jane was awaiting genetic testing for the same. A family history of autism spectrum disorder (ASD), attention deficit hyperactivity disorder (ADHD) and epilepsy were also noted in Jane's sibling. Jane's maternal grandmother had a history of severe mental illness of unknown diagnosis. Jane had a history of migraines but no other medical history or known neurodevelopmental conditions. Jane was referred for an autism assessment due to social communication difficulties and sensory sensitivities. A diagnosis of ASD was subsequently made. The multi‐disciplinary team agreed on an initial working diagnosis of moderate depression and recommended for Jane to receive individual cognitive behavioural therapy (CBT) and group dialectical behaviour therapy (DBT).

Jane's symptoms worsened in October 2023 following starting a new school, a significant change. She experienced increased anxiety, worsening mood with agitation, and escalating suicidal ideation. In November 2023, at her psychiatric review, Jane expressed a desire to trial an anti‐depressant. A plan was made for a slow titration of sertraline and to discuss with neurology if a recent prescription of sumatriptan could be switched to another anti‐migraine medication, given the risk of serotonin syndrome when co‐prescribed. Jane discontinued sumatriptan due to nausea and began sertraline 3 days later, initially at 25 mg once a morning for 1 week, increasing to 50 mg thereafter.

2 weeks later Jane was followed up (on sertraline 50 mg) and reported no improvement in her low mood. Jane, however, reported worsening sleep, heightened anxiety, derealisation, and vague experiences of hearing her name called in her room (without psychotic conviction). No changes to her medication were made. 3 days later, a change in Jane's presentation was noted. She had absconded from school, reporting new grandiose plans and was later found by the police at a bridge. At her psychiatric review, Jane reported having increased energy, feeling ‘high’ in mood, and shared her grandiose plans. She presented as distractible and, although there was ongoing sleep disturbance, Jane was not tired. Her Young Mania Score rated 13. Due to concerns of emerging hypomanic symptoms, sertraline was discontinued and Jane was prescribed promethazine 25 mg at night to aid sleep.

**FIGURE 1:**
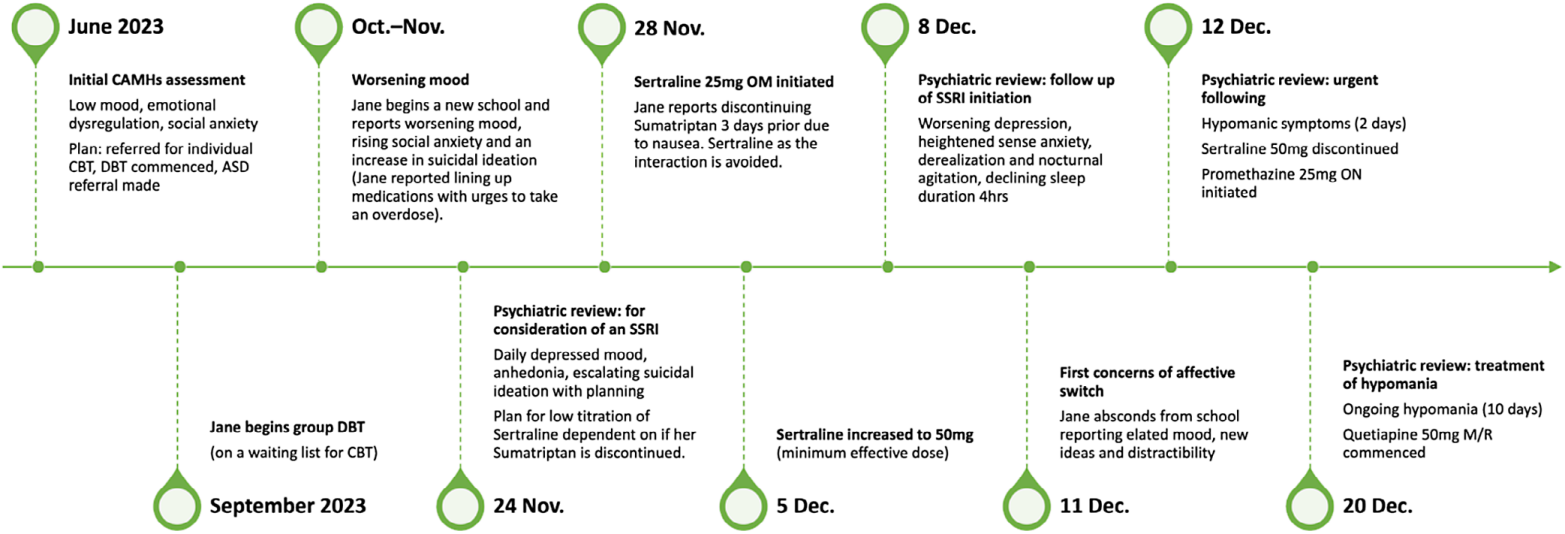
Timeline of Jane's worsening depression and affective switch following sertraline initiation.

At a further psychiatric review 1 week later, Jane continued to experience an expansive mood, irritability with aggressive outbursts, unusual experiences, and poor sleep. Although Jane was on school holidays, she could function socially and in necessary activities of daily living. Following an electrocardiogram with normal results, Jane was started on Quetiapine modified release 50 mg at night. At the time of writing, Jane's symptoms are ongoing (over 30 days following cessation of sertraline). The current plan is to increase Quetiapine MR to a therapeutic dose for bipolar disorder. Jane has also started CBT for psychosis as this includes psychoeducation for bipolar disorder and work around sub‐threshold perceptual abnormalities. A timeline of events can be found on Figure 1.

## 2 Discussion

Jane experienced a hypomanic episode after treatment with sertraline which persisted beyond cessation, with pharmacological intervention being necessary to treat symptoms. Given her background of recurrent depression, her case raises both diagnostic and clinical considerations. Firstly, is Jane's hypomania indicative of an underlying “endogenous” bipolar type II disorder (BD‐II), or should her symptoms be considered treatment emergent? Secondly, dependent on the relationship between these concepts, how should clinical management proceed?

Several terms have historically been used to categorise individuals who experience mania or hypomania in the context of medical treatments. Notably Akiskal classified individuals as “Bipolar III” if they experienced hypomania with anti‐depressants [1], whereas a taskforce of the international society of bipolar disorder (ISBD) operationalised “definite” treatment emergent affective switches (TEAS) as mania/hypomania lasting at least 2 days and occurring within 8 weeks of a change to a known precipitating medication (anti‐depressants or somatic treatments) [2]. While these terminology and criteria have not been directly incorporated into current diagnostic classification systems, adjacent categories do exist, namely ‘substance/medication‐induced bipolar disorder’ in the diagnostic and statistical manual of mental disorders (DSM) version‐V, referring to mania/hypomania that arises during intoxication or withdrawal of an illicit substance or medication. Nonetheless, other than serving to describe specific mood episodes, these labels do not provide insight into the causality of treatment emergent symptoms, and how an individual with a first presentation of TEAS may relate or differ to another with primary bipolar affective disorder. For Jane, the nature of this relationship is crucial as it would gauge the likelihood of a future “spontaneous” mania/hypomania, thereby indicating if maintenance treatment would be beneficial. However, assessing whether an anti‐depressant is the precipitant of hypomania can be challenging, due to shared symptomatology across affective states and overlapping timelines.

Anti‐depressants are often started when patients are most depressed and may be experiencing symptoms that could also be present during a hypomanic prodrome such as insomnia. For Jane, Sertraline started during a period of sleep deprivation and nocturnal agitation, generating uncertainty on whether these features were indicative of an already emerging affective switch that may have occurred regardless of anti‐depressant initiation. While the ISBD criteria for TEAS specifies an affective switch is considered “definite” should it occur within 8 weeks of anti‐depressant treatment [2] this broad range does not indicate the degree to which anti‐depressant treatment contributed to the switch over and above any underlying bipolarity. Malhi et al. [3] suggest a shorter time lag may increase confidence that anti‐depressant treatment is primarily implicated in emergence of manic symptoms, and additionally that other features such as the rate of symptom emergence, the severity of mania/hypomania, and the extent of recovery following anti‐depressant cessation can be used to indicate the extent that underlying bipolarity contributed to TEAS [3]. Applying this guidance to Jane would suggest an elevated vulnerability to hypomania and a closeness to bipolar disorder, given that her hypomanic symptoms persisted despite cessation of sertraline. DSM‐V indicates that if symptoms remain at full syndrome level following the physiological effects of a medication have worn off then a primary manic or hypomanic episode can be diagnosed. However, if mood elevations only occur in the context of anti‐depressant treatment can we confidently diagnose a bipolar‐II disorder?

In adolescents there is less certainty about the causal role of anti‐depressants in affective switches. A recent large retrospective cohort study by Virtanen et al. [4] found anti‐depressant treatment did not increase incidence of mania/hypomania at 12‐weeks in adolescents with unipolar depression. Instead, other predictors such as past hospitalisation, a family history of bipolar disorder or psychotic symptoms were associated to an increased incidence of mania/hypomania at extended follow up (52‐weeks), suggesting that anti‐depressants themselves may only be initiated in a subgroup of depressed teenagers. However these findings contrast to an earlier register based study which linked anti‐depressant initiation to an increased risk of mania/hypomania, here assessed at 52‐weeks Martin et al. [5].

Where uncertainty arises about the degree that anti‐depressant treatment has contributed to an adolescent's affective switch, and if a bipolar diagnosis can be given, how should clinicians interpret treatment emergent hypomanic symptoms? Utilising the bipolar‐at‐risk framework the authors suggest using treatment emergent affective symptoms as a warning sign of underlying bipolarity, thereby promoting a period of increased monitoring to identify early transition to bipolar disorder.

While some diagnostic latency may be expected due to the changing clinical presentation during puberty and age‐related social transition, there remains a need for earlier identification of bipolar disorder. Akin to psychotic disorder expanding these early intervention services for bipolar disorder may help mitigate negative outcomes.

## Ethics Statement

As this case report gained informed consent and used anonymised patient information it did not require ethical consent via an Institutional Review Board.

## Conflicts of Interest

The authors declare no conflicts of interest.

## Data Availability

The authors have nothing to report.
